# Diffuse benign inflammatory gastric polyps: A rare case in a young female: Case report and review of the literature

**DOI:** 10.3389/fsurg.2022.1090622

**Published:** 2023-01-05

**Authors:** ZongYao Zhang, ZhengChang Guo, JiaJia Zhang, Xin Yu

**Affiliations:** ^1^ Department of General Surgery, The First Affiliated Hospital of Anhui University of Science and Technology, Huainan, China; ^2^ Department of General Surgery, Zhenjiang First People's Hospital, Zhenjiang, China; ^3^ Department of General Surgery, The First Affiliated Hospital of Anhui Medical University, Hefei, China

**Keywords:** diffuse gastric polyps, inflammatory polyps, case report, surgical intervention, diagnosis

## Abstract

**Background:**

Gastric polyps are one of the most common clinical diseases arising from the mucosal surface of the stomach. The benign nature of the gastric polyp and its absence of symptoms have been widely accepted. Diffuse benign inflammatory polyps spanning the entire gastric mucosa are relatively rare in young people.

**Case presentation:**

Our objective was to report a 20-year-old woman who presented with epigastric pain and vomiting; upper gastrointestinal barium contrast roentgenography demonstrated a huge defect in the filling of the stomach. Upper endoscopy also showed the presence of dense inflammatory polyps in the stomach that were the cause of the severe pylorus obstruction. The diffuse benign gastric polyps were diagnosed as inflammatory gastric polyps on the basis of findings on the histopathological examination. She was delivered as a result of the operating procedure of total gastrectomy and Roux-en-Y anastomosis of the esophagus and jejunum. Postoperative nutritional support therapy was also implemented. Postoperative pathological examination revealed inflammatory papillary and villous polyps distributed over the stomach, and eosinophilic infiltration was found in the local area of the polyp. Polyps move like tufts of coral. During the 16-month follow-up, patients with symptoms of malnutrition and anemia recovered.

**Conclusion:**

Nutritional support and a total gastrectomy were used to improve this patient's symptoms of malnutrition and anemia. Surgical intervention with appropriate nutritional support should be actively performed in these patients while strengthening the differential diagnosis of hereditary disease.

## Introduction

Gastric polyps (GPs) are sessile or stalked lesions that can be traced to the gastric epithelium and submucosa and, at certain times, may even protrude into the stomach (1). Inflammatory gastric polyps are widely recognized as one of the common diseases encountered in the clinic. However, the diffuse benign inflammatory polyps in the stomach are rarely reported in recent decades. In people over the age of 60, inflammatory gastric polyps often occur throughout the gastrointestinal tract (2). The purpose of this case report is to describe the case of a young female patient with gastric polyps who was hospitalized for anemia and pyloric obstruction. Gastric polyposis also occurs in patients with a variety of gastrointestinal polyposis syndromes (3). We initially performed relevant tests, treatments, and genetic testing, but the patient cannot be diagnosed with any type of polyp syndrome. Finally, we performed surgery and enteral nutrition as part of the treatment.

## Case presentation

We had a 19-year-old woman admitted to our hospital because she complained of epigastric pain for nearly 2 years, and vomiting and fatigue for 3 months, with a weight loss of 10 kg over the last 8 months. At one time, the patient was considered to have chronic gastritis at the local hospital in her hometown. ibuprofen and omeprazole therapy strategy for 3 months was performed, although there was no relief of gastrointestinal symptoms. The patient was a student with an unremarkable history.

**Figure 1:**
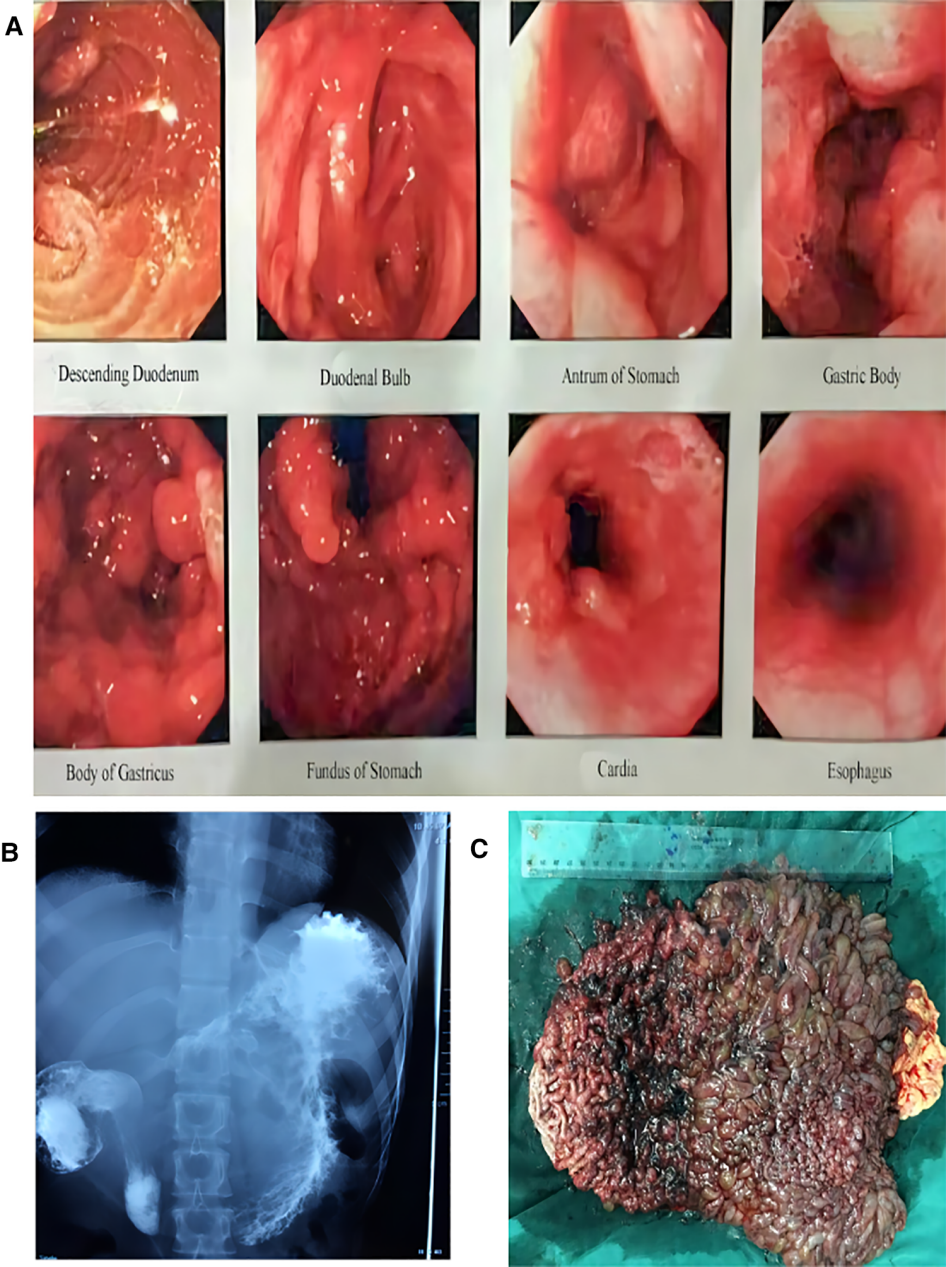
(**A**) Endoscopic findings. An upper endoscopy showing the diffuse benign inflammatory gastric polyps caused a subtotal obstruction of the gastric outlet. (**B**) UGBCR showed gastric mucosal destruction and a huge filling defect in the stomach. (**C**) The stomach is being filled with polyps. These polyps showed a coral plexiform change. UGBCR, upper gastrointestinal barium contrast roentgenography.

There is no family history of gastrointestinal polyposis in the patient. Physical examination findings included anemia, upper abdominal tenderness (+), and loss of body weight (body weight: 40 kg). In addition, positive cardiac, pulmonary, and limb findings were found. Laboratory results revealed a hemoglobin level of 90 g/L. The biochemistry of the blood was potassium 2.66 mmol/L, sodium 130.8 mmol/L, chlorine 75.4 mmol/L, and albumin 31.5 g/L, whereas the rest of the blood biochemistry results were normal. Upper endoscopy demonstrated numerous finger-like polyps and nodular lesions that aggregated in the antrum, duodenal bulb, and extension of the gastric body into the cardia (Figure 1A). Upper gastrointestinal barium contrast roentgenography (UGBCR) demonstrated that gastric mucosal destruction and an enormous filling defect were located in the stomach (Figure 1B). Diffuse benign inflammatory gastric polyps also had significantly reduced gastric cavity volume. These polyps resulted in a subtotal gastric outlet obstruction. A single balloon enteroscopy was performed, and two were found in the ileocecal area and descending colon. Nodular polyps were 0.5–1 cm in size, with a mean of 0.5 cm. To obtain more histopathologic information, the patient, her siblings, and relatives were screened for relevant oncogenes and gastrointestinal tumor biomarkers. Thus, no clear mutations in related genes or abnormal indicators were identified. Furthermore, gastroscopy findings from the patient's parents and brother did not reveal any obvious abnormalities.

Upon review of all clinical findings, we initially diagnosed this patient as having diffuse gastric polyps with pyloric obstruction, moderate anemia, hypoproteinemia, and an electrolyte imbalance. We then placed the jejunal nutrition tube under the endoscope to the inferior duodenum and performed extensive enteral and parenteral nutrition therapy over the course of 1 month. After 1 month, a total gastrectomy and Roux-en-Y anastomosis were performed. At the time of surgery, the stomach was markedly dilated and filled with polyps (Figure 1C). There was a slight dilatation of the descending portion of the duodenum. In addition, no obvious masses were affected in the small bowel and colorectum. On histopathologic examination, the gastric cavity was found to be covered with inflammatory villous and papillary polyps with a total extent of 2.3 cm × 1.6 cm. A coral plexiform change was observed in the polyps, with a protrusion length ranging from 1 to 6 cm and a diameter ranging from 1.3 to 2.0 cm.

**Figure 2:**
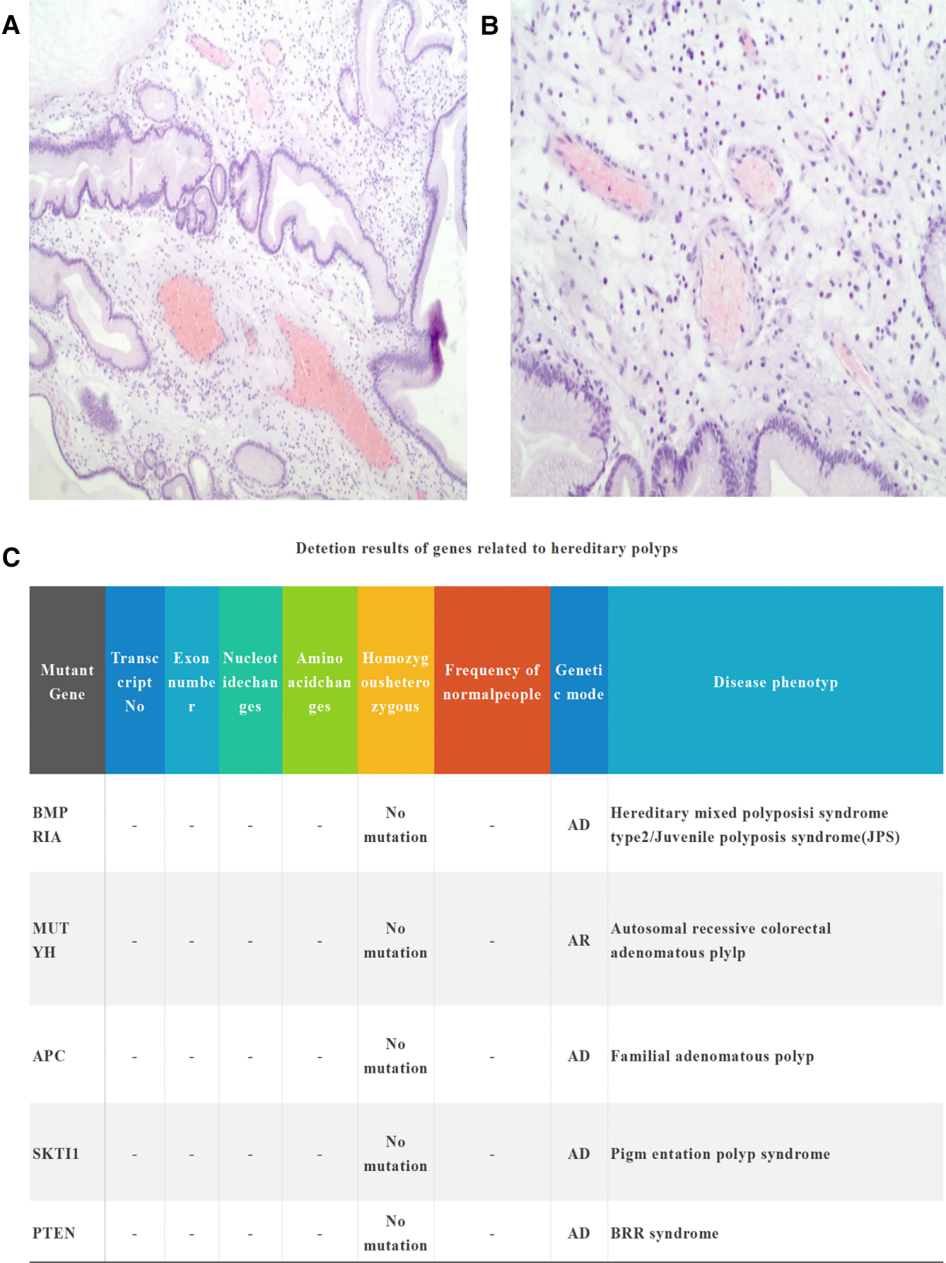
(**A**) Histopathological view through a medium-power microscope. Microscopic findings reveal the infiltration of eosinophils cells. (**B**) Polyp lesions do not invade the muscularis (hematoxylin–eosin ×100). (**C**) Hereditary polyposis syndrome and malignancy-associated genes are negative.

The lesions of the polyps do not invade the muscularis muscle. There was still a clear boundary between the mucosal layer and the muscularis layer; in addition, infiltration of eosinophils into the local portion of the polyp was observed. On lateral examination, 26 lymph nodes next to the stomach showed reactive hyperplasia. In addition, microscopic findings revealed papillary villous inflammation polyp (Figures 2A,B). At 12-month postoperative follow-up, the patient's nutritional status had significantly improved with an increase in weight of up to 10 kg.

## Discussion

The gastric polyp arises from the surface of the gastric mucosa. Pathological types include inflammatory, hyperplastic fundic gland, adenoma, hamartoma, and juvenile gastric polyps (4). Conventional types of gastric polyps have different prevalences in different parts of the world. In previous studies, hyperplastic gastric polyps have been reported to be the most common polyps in Western countries, followed by inflammatory polyps (5). Likewise, the most common histologic types are also inflammatory polyps and hyperplastic polyps in China (6). Most of the inflammatory surface of gastric polyps is smooth in clinical and pathological features, but a few parts go hand in hand with ulcerative erosion. It consists mainly of inflammatory cells and inflammatory granulation tissue without congland, has a low cancer rate, and appears mostly in the gastric body. Gastroscopy or follow-up clinical observation is the mainstay of clinical treatment (7). At present, the pathogenesis of inflammatory polyps is indistinct, with several research reports suggesting that its pathogenesis is linked to *Helicobacter pylori* and chronic inflammatory infections (8). In the current study, infection with *H. pylori* promoted an inflammatory response *via* interleukin-1 and hepatocyte growth factors, leading to gastric epithelial cell proliferation and thus inflammatory polyp formation (9).

Although diffuse benign gastric polyps (more than 100 pcs) are quite rare in the clinic, in this case, other syndromes of hereditary polyposis and malignancy need to be excluded, such as familial adenomatous polyposis, Peutz–Jeghers syndrome (PJS), juvenile polyposis syndrome (JPS), and hair loss Cronkhite–Canada syndrome (CCS). . The rate of malignancy of diffuse benign gastric hyperplasia in the incidence of polyp lesions is up to 3.6% (10). Familial atheromatous polyposis (FAP) is an inherited cancer syndrome that includes gastrointestinal involvement, polyposis, and the potential for adenocarcinoma to develop. Common symptoms include hamartomas, hyperplastic polyps, and adenomas in FAP, fundic gland polyposis for the most part (51%). Histologic types include tubular adenomas, villous tubular adenomas, or villous adenomas in the FAP (11). Tubular adenoma is the most common and villous adenoma is rare with high rates leading to cancer. Despite the rarity of gastric malignancy in patients with FAP, it is still difficult to diagnose it as a premalignant change solely on the basis of endoscopy symptoms (12). PJS is a rare autosomal dominant disorder that has been characterized by hamartomatous polyps throughout the gastrointestinal tract, most commonly causing gastrointestinal bleeding and recurrent bowel obstruction (13). JPS is a sporadic disease consisting of numerous hamartomatous polyps found throughout the gastrointestinal tract in patients, particularly in the colon. Patients with juvenile polyposis syndrome are required to have more than five juvenile colorectal polyps, a number of juvenile polyps in the remnant gastrointestinal tract, and a family history of JPS (14). CCS is a rare, noninherited disease that has been characterized by gastrointestinal polyposis and ectodermal defects including hyperpigmentation, alopecia, and onychodystrophy (15). In the present case, the patient had no known familial tendency for skin or mucosal pigmentation. Seventy-one genes were found to be mutant genes based on genetic testing, and 100% were normal. Inherited syndromes of polyposis and genes for malignancy were negative (Figure 2C). No mutations in related genes were observed. In our patient, dense gastric polyps were confined to the stomach and did not appear to fit into any of these diffuse polyposis syndromes.

In most reports of diffuse benign inflammatory gastric polyps, the differences between inflammatory polyps and polyposis due to hyperplasia do not exist. There are few case reports of diffuse benign gastric polyps. Hu et al. reported a case of diffuse gastric polyposis brought on by pain in the lower abdomen and anemia (16). The authors reported that diffuse gastric polyps are highly indicative of diffuse hyperplastic polyps without carcinoma. Chongsrisawat et al. also mentioned a case of a 4-year-old girl with abdominal pain who was considered to have diffuse inflammatory gastric polyp (17). Jayawardena et al. cloaked one case of a 26-year-old woman who presented with multiple onset polyps due to severe anemia (18). Similarly, (20) reported a 50-year-old woman with diffuse gastric polyposis who presented with anemia. Jaka concluded that a 19-year-old male patient had diffused benign inflammatory gastric polyps with acute gastrointestinal bleeding and anemia (19). Table 1 displays our case summary of diffuse benign gastric polyposis in recent years because there are no relevant case reports. Therefore, we suggest having joint multidisciplinary consultations to identify related genetic conditions and conduct genetic testing at the same time, which will assist in determining appropriate therapy.

**Table 1:** Literature review of diffuse benign gastric polyps.

Country	Age	Sex	Treatment	Symptoms	Outcome	Reference
China	59	Female	Total gastrectomy	Lower abdominal and anemia	Survived	Hu et al. (2002)
Thailand	4	Female	Distal gastrectomy	Abdominal pain	Survived	Chongsrisawat et al. (2004)
United States	26	Female	Total gastrectomy	Profound anemia	Survived	Jayawardena et al. (2008)
Italy	50	Female	Total gastrectomy	Anemia	Survived	Spaziani et al. (2011)
Tanzania	19	Female	Total gastrectomy	Gastric blood	No known	Jaka et al. (2013)

The patient in this case did not have any distinctive clinical symptoms or manifestations for 2 years. The gastric polyps were progressively enlarged during this time. Due to a lack of understanding of the cause of the condition and clinical diagnosis, conservative internal medicine treatment is not successful. The patient gradually developed symptoms of pyloric obstruction and severe malnourishment. Given that this disease is uncommon clinically and is frequently accompanied by complications such as anemia, gastrointestinal bleeding, and congestion, we should combine surgical intervention with active nutritional treatment. Patients with diffuse gastric polyps who present with anemia and pyloric obstruction may benefit from nutritional support in terms of nutritional status and quality of life. Surgeries include partial gastrectomy as well as total gastrectomy. In many ways, we need to think about both the clinical effect and the trauma to the patient's body, as well as postoperative complications. Younger patients will have long-term complications after total gastrectomy such as reflux esophagitis, malignant anemia, malnutrition, and so on. Follow-up gastrointestinal and colonoscopic surveillance should be performed regularly after surgery. Gastroscopy can be convenient to perform in most patients and is seen in unusual and complicated cases that need prompt diagnosis. The cause of the rapid growth of polyps in this patient is not clear and needs further exploration and research to be clarified. We conclude with a case report of diffuse benign inflammatory gastric polyps in a young female patient.

## Conclusion

We should actively strengthen the differential diagnosis of hereditary gastrointestinal polyp syndrome and malignant potential based on the case presented here. In addition, nutritional support and total gastrectomy can be used to improve those patients' symptoms of malnutrition and anemia. Surgical intervention with appropriate nutritional support should be actively performed in these patients.

## Data Availability

The original contributions presented in the study are included in the article/Supplementary Material, further inquiries can be directed to the corresponding author.
